# The ‘SAR Matrix’ method and its extensions for applications in medicinal chemistry and chemogenomics

**DOI:** 10.12688/f1000research.4185.2

**Published:** 2014-06-23

**Authors:** Disha Gupta-Ostermann, Jürgen Bajorath

**Affiliations:** 1 Department of Life Science Informatics, B-IT, LIMES Program Unit Chemical Biology and Medicinal Chemistry, Rheinische Friedrich-Wilhelms-Universität, Bonn, D-53113, Germany

## Abstract

We describe the ‘Structure-Activity Relationship (SAR) Matrix’ (SARM) methodology that is based upon a special two-step application of the matched molecular pair (MMP) formalism. The SARM method has originally been designed for the extraction, organization, and visualization of compound series and associated SAR information from compound data sets. It has been further developed and adapted for other applications including compound design, activity prediction, library extension, and the navigation of multi-target activity spaces. The SARM approach and its extensions are presented here in context to introduce different types of applications and provide an example for the evolution of a computational methodology in pharmaceutical research.

## Introduction

Steadily growing numbers of active compounds provide a critically important knowledge base for medicinal chemistry but also challenge Structure-Activity Relationship (SAR) analysis ^1^. For important therapeutic targets, compound activity landscapes become increasingly complex ^2^ and difficult to analyze. Increasing volumes and complexity of compound activity data require the development of computational approaches to effectively extract SAR information from heterogeneous sources ^1^. In addition, it is essential to make this information available in an intuitive form that can be appreciated in the practice of medicinal chemistry and utilized in compound design. Therefore, a number of SAR visualization methods and graphical analysis tools have been developed in recent years ^2, 3^ to view SAR characteristics of entire data sets or extract SAR information from compound activity data. Regardless of their algorithmic foundations and design specifics, many (but not all) graphical analysis methods have in common that they provide a bird’s eye view of SAR information in compound data sets and depart from the single-series focus that has traditionally governed medicinal chemistry efforts. However, multi-facetted SAR information obtained from heterogeneous compound sources must ultimately again be utilized to advance individual compound series, which is a challenging task.

The Structure-Activity Relationship Matrix (SARM) approach has originally been designed to extract and organize SAR-informative compound series from large data sets ^4^ and has been further extended to help bridge the gap between data-driven SAR analysis, compound design, and activity predictions ^5^ and study compound series in multi-target activity spaces ^6^. Here, we present the SARM approach and its extensions in context and introduce new features and applications.

## Methods

### Compound structure analysis and organization

The original design idea underlying the SARM approach was to systematically extract compound series with well-defined structural relationships from data sets and organize them in a matrix format ^4^. To convey SAR information, matrix cells representing data set compounds are color-coded according to compound potency. The methodological basis for compound series identification and organization was provided by the matched molecular pair (MMP) concept ^7^. An MMP is defined as a pair of compounds that differ only at a single site ^7^. Compounds in MMPs can be interconverted by the exchange of a substructure, termed a chemical transformation ^8^. In order to generate MMPs on a large scale, compounds must be systematically fragmented. The algorithm by Hussain and Rea ^8^ (which we re-implemented and further modified in-house) provides an elegant and computationally efficient solution to this task by subjecting compounds to systematic deletion of individual exocyclic single bonds (single-cut) or simultaneous deletion of two (dual-cut) and three (triple-cut) exocyclic single bonds. The resulting fragments are then stored in an index table as keys (core structures) and smaller values (substituents) ^8^.

**Figure 1.**
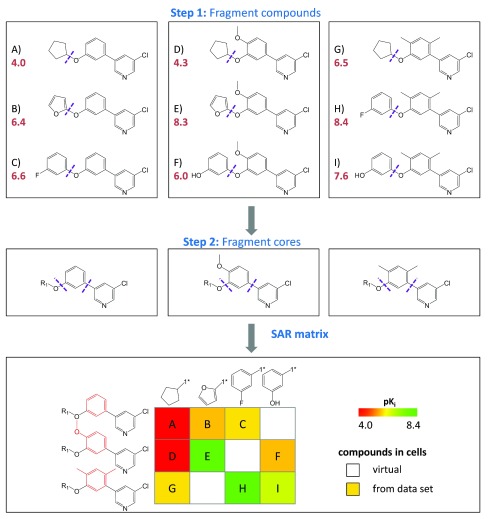
SAR matrix generation. Three model series with three compounds each ( **A**– **C**, **D**– **F**, and **G**– **I**) are shown with pK _i_ values (red). In the first step, all compounds are fragmented at a single bond (purple dotted line) producing compound MMPs that yield a common core (key) and a compound specific substituents (values). In the second step, the cores resulting from the first step are further fragmented to obtain core MMPs. The SARM is then generated by combining series with structurally analogous cores that represent individual rows. In addition, columns represent substituents. In each cell, the combination of a core and a substituent defines a unique compound. Compounds present in the data set are indicated by filled cells that are color-coded according to potency using a continuous spectrum from red (low potency) over yellow to green (high). In addition, empty cells indicate virtual compounds. Substructures distinguishing the core fragments are highlighted in red.

The most important aspect of SARM design has been the application of dual fragmentation scheme leading to MMP generation at two levels ^4^, as outlined in Figure 1. In the first step, MMPs are generated from data set compounds yielding “compound MMPs”. In the second step, core fragments from compound MMPs are again subjected to fragmentation leading to the generation of “core MMPs”. As a consequence, this hierarchical two-step fragmentation scheme identifies all compound subsets that have structurally analogous cores, i.e., core structures that are only distinguished by a structural modification at a single site. Each subset represents a so-called “structurally analogous matching molecular series” (A_MMS) ^4^. Thus, each A_MMS represents a set of compound series with structurally analogous cores. Individual compounds and/or subsets of compounds can belong to multiple A_MMS, hence providing a high-level structural organization of a compound collection that captures all possible (MMP-based) substructure relationships.

### SAR matrix design

Each A_MMS is represented in an individual SARM, as illustrated in Figure 1. The SARM is filled with structurally analogous cores resulting from core MMPs (second fragmentation step) and the corresponding substituents obtained from compound MMPs (first fragmentation step). Single-, dual-, and triple-cut matrices are separately generated ( *vide supra*). Each cell in a SARM represents a unique compound, i.e., a unique combination of a key and value fragment. Each row contains an individual analog series, i.e., compounds sharing the same core. Each column contains compounds from different series that share the same substituent (single-cut) or substituent combination (dual- or triple-cuts). The series forming a SARM typically contain different sets of substituents, giving rise to “real” compounds (filled cells) and “virtual” compounds (VC; empty cells). As also illustrated in Figure 1, a color spectrum is applied to represent the potency (or ligand efficiency) values of real compounds. Importantly, SARMs resemble standard R-group tables used in medicinal chemistry, although their design and information content is much more complex and comprehensive. Standard R-group tables typically only contain an individual core structure of a single series, all substituents, and associated potency values. However, because SARMs resemble R-group tables, they are readily accessible to medicinal chemists who can inspect individual compounds and their relationships to others.

### SAR patterns

**Figure 2.**
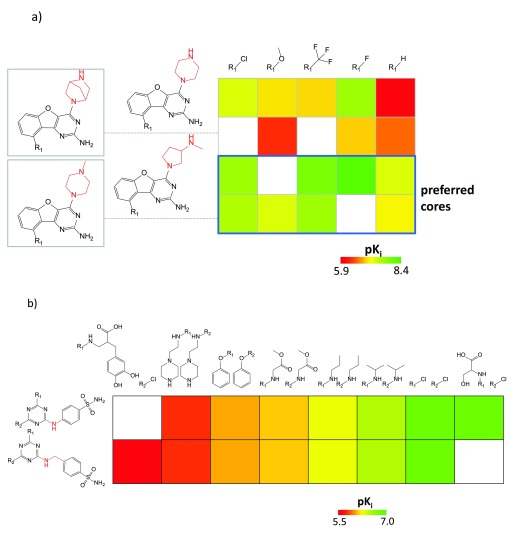
SAR patterns in matrices. In ( **a**), a SARM capturing 16 ligands of the histamine H4 receptor is shown. Cells containing analogs with preferred cores yielding potent compounds are framed in blue. Substructures distinguishing the core fragments are highlighted in red. The pK _i_ value range for the 16 ligands is displayed. In ( **b**), a subset of a double-cut SARM is shown that contains series of carbonic anhydrase I inhibitors and an exemplary SAR transfer event.

In SARMs, different types of SAR patterns become readily apparent. This is illustrated in Figure 2 that shows exemplary SARMs revealing characteristic patterns (for representation purposes, only small matrices are shown; *vide infra*). For example, the SARM in Figure 2a identifies two preferred core structures that consistently produce potent compounds. Furthermore, the SARM in Figure 2b reveals an SAR transfer event, i.e., the presence of two compound series with related yet distinct core structures that contain pairwise corresponding analogs with similar potency progression. Other SAR patterns that can frequently be detected include, for example, preferred R-groups (or R-group combinations) in related compound series or regions of distinct SAR continuity or discontinuity. Continuous SAR regions are characterized by the presence of compounds with structural modifications that lead to gradual changes in potency, whereas discontinuous SAR regions contain structural analogs with large (and essentially unpredictable) potency variations ^2^.

### Matrix distribution and ranking

**Figure 3.**
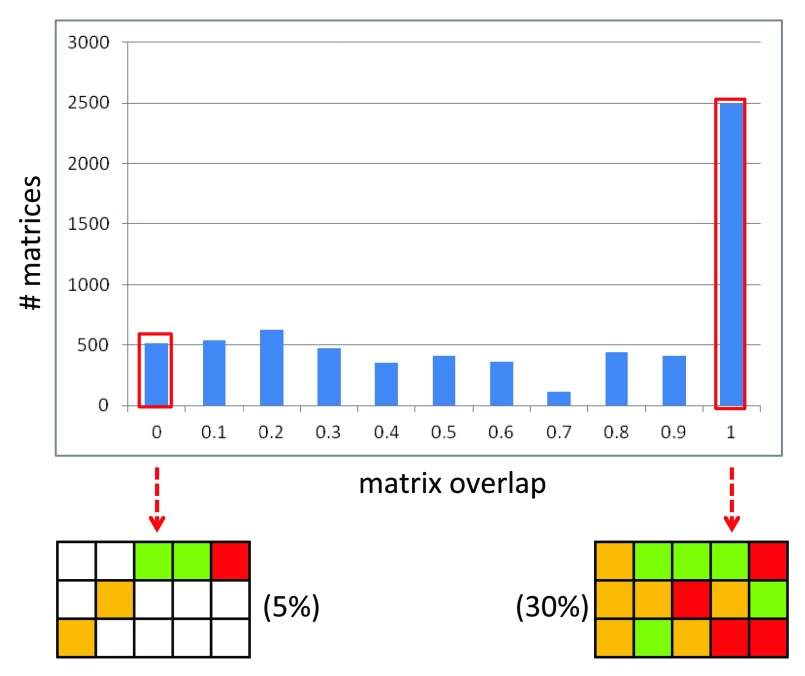
Matrix overlap distribution. Shown is a histogram with the matrix overlap distribution for SARMs from an in-house focused library.

Large compound data sets typically yield many SARMs of different size and composition, depending on their degree of structural homogeneity or heterogeneity. Two examples are given to illustrate this point. First, an in-house focused compound library with various substitutions of a small number of core structures comprising 6503 compounds produced a total of 6738 (single-, double- and triple-cut) matrices containing a total of 135,619 VCs. Second, a structurally heterogeneous set of 509 purinergic receptor (P2Y12) ligands generated a total of 181 SARMs containing 17,445 VCs. Again, each SARM contains a unique A_MMS and individual compounds might belong to multiple A_MMS depending on the structural relationships they form. SARMs provide highly resolved views of all of these structural relationships. Depending on the number of compounds forming A_MMS, the size of SARMs can considerably vary. For example, in a survey of 32 different activity classes consisting of 398 to 2497 compounds, SARMs were found to contain between three and 555 compounds, with a median value of 13. Furthermore, we also use a “matrix overlap” measure to account for the overlap between the corresponding substituents (columns) in different A_MMS (rows), which typically varies in SARMs. Matrix overlap is determined as the average over all row overlap values. For individual columns in SARMs, row overlap (RO) is calculated as:

$$\text{RO}=\frac{n\_col-\text{1}}{\#rows-1}$$

where, *n_col* correspond to the number of data set compounds present in each column. RO yields a numerical score between 0 (no overlap) and 1 (complete overlap). Figure 3 reports the matrix overlap distribution for SARMs from the focused library referred to above, which is a fairly representative distribution for structurally homogeneous data sets. Here 5% of the SARMs have an RO of 0 for each column; hence, the final matrix overlap score is 0 indicating the mutually exclusive nature of the substitution pattern among the A_MMS. By contrast, 30% of the SARMs have an RO of 1 for each column; hence, the final matrix overlap is 1 reflecting the presence of A_MMS with identical substitution patterns. As an alternative measure, “matrix coverage” (C), which accounts for the proportion of cells in a SARM that are populated with real compounds *n_matrix* can be calculated as:

$$\text{C}=\frac{n\_matrix}{\#rows*\#columns}$$

**Figure 4.**
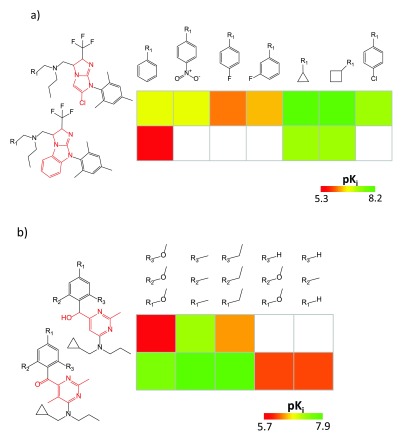
Ranked SAR matrices. In ( **a**) and ( **b**), two SARMs are shown (resulting from single- and triple-cut fragmentation, respectively) for corticotropin-releasing factor receptor 1 ligands that were highly ranked on the basis of SAR discontinuity scoring.

Regardless of the number of SARMs that are obtained from large data sets, there are too many for one-by-one inspection. Hence, ranking schemes should be applied to prioritize and pre-select those SARMs that are most informative for a given application. For instance, SARMs can be easily ranked on the basis of numerical functions that prioritize matrices containing preferred substituent combinations or core structures and SAR transfer events or matrices that capture high degrees of local SAR continuity or discontinuity. For example, Figure 4 shows two SARMs originating from a large data set that are highly ranked on the basis of SAR discontinuity (as indicated by the presence of multiple analogs with large potency differences). Depending on the applied selection criteria, most informative SARMs can be readily inspected on the basis of a ranked list.

### Compound design and activity prediction

**Figure 5.**
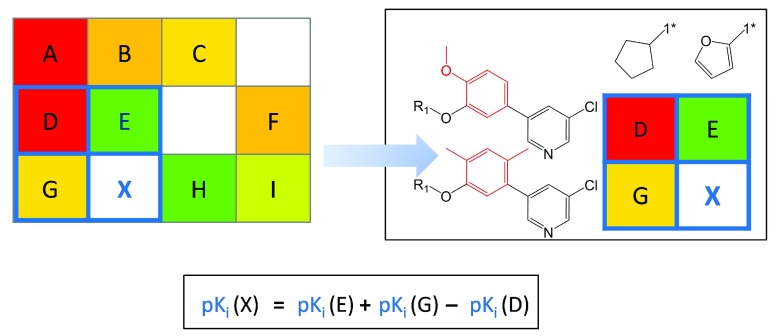
Neighborhood-based potency prediction. An NBH of virtual compound X is marked in blue in a model SARM and displayed in detail. Compounds E and G share the same substituents and core with X, respectively, and the third neighbor D combines the core and substituent of E and G, respectively. At the bottom, the equation to predict the potency of X from the potency values of E, G, and D is shown.

VCs contained in SARMs provide immediate suggestions for compound design. Because VCs represent unexplored key-value combinations derived from data set compounds, the union of VCs from all SARMs provides a “chemical space envelope” for a given compound set or library. VCs originating from SAR-informative matrices represent natural focal points for interactive compound design. Moreover, the potency of many virtual compounds can be predicted by applying a compound neighborhood (NBH) principle ^5^, as illustrated in Figure 5. An NBH of a given VC is defined by three adjacent real compounds that contain the core of the VC (compound G in Figure 5), its substituent (compound E) and the core and substituent of G and E (compound D). The potency of the VC can then be predicted by applying the additivity assumption underlying Free-Wilson analysis ^9^ using the simple equation shown in Figure 5. The putative potency value of the VC results from the sum of (logarithmic) potencies of the two real compounds sharing the same core and substituent with the VC, respectively, minus the potency of the compound that contains the core structure and substituent of the two other real compounds. Thus, from NBHs, “mini-QSAR” models are derived for activity prediction. For each candidate VC, qualifying NBHs are collected across all SARMs, individual potency predictions are carried out, and their consistency is evaluated, for example, by calculating standard deviations for predictions ^5^. In benchmark calculations on six different sets of G protein-coupled receptor ligands, potency values of subsets of test compounds falling into continuous local SAR regions were accurately predicted using the NBH-based approach, and prediction accuracy generally increased with the number of qualifying NBHs ^5^. This is also relevant for practical applications. For potency prediction, candidate VCs should be prioritized for which multiple NBHs are available. For example, for the set of 509 purinergic receptor ligands ( *vide supra*), 5167 of 17,445 VCs were found to have at least three qualifying NBHs. Hence, in these cases, the consistency of potency predictions can be assessed. Such candidate VCs can be explored in a systematic manner. For libraries tested in individual assays, VCs predicted to be consistently active on the basis of multiple NBHs provide preferred candidates for target/assay-dependent library expansion and focusing.

**Figure 6.**
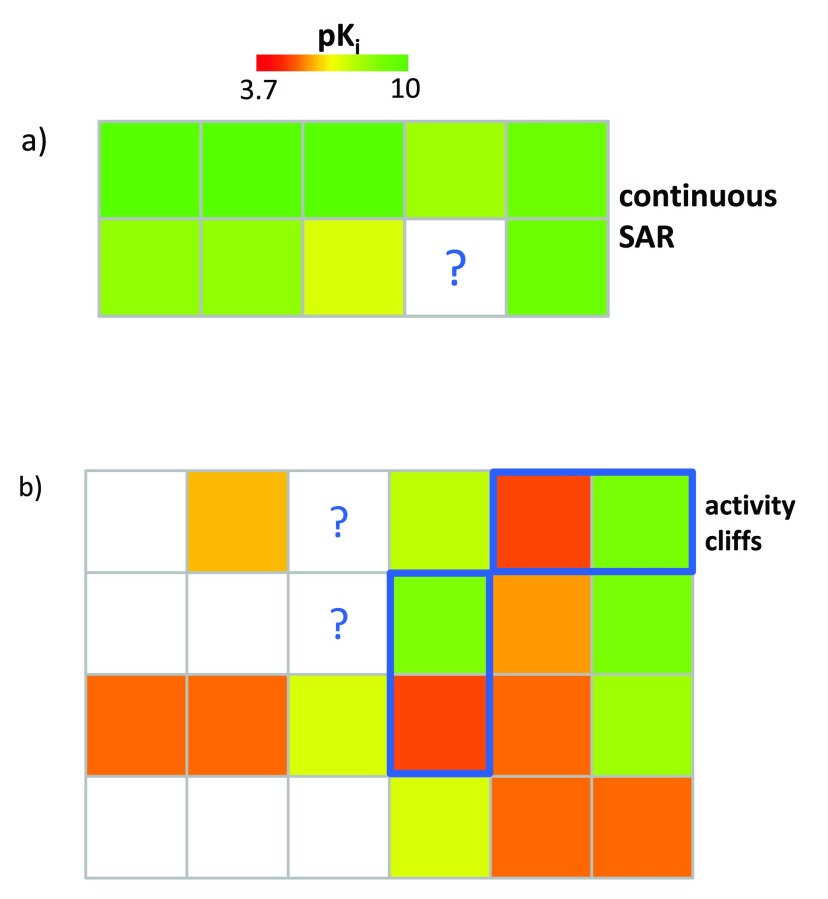
Candidate compound selection and activity prediction. In ( **a**), a SARM is shown that represents a highly continuous local SAR environment. In this case, the potency of a virtual compound can be predicted using the NBH-based approach. By contrast, ( **b**) shows a SARM representing a discontinuous local SAR. Activity cliff-forming compound pairs are highlighted in blue. Such SAR environments fall outside the applicability domain of NBH-based potency predictions. However, marked VCs represent promising candidates for compound design based on their proximity to activity cliffs. Both SARMs originate from a set of cannabinoid CB1 receptor ligands (compound structures are omitted for clarity).

Importantly, the NBH-based mini-QSAR approach is only applicable to candidate compounds falling into SARMs that represent continuous SAR regions, as illustrated in Figure 6a. By contrast, compounds falling into discontinuous SAR regions, as shown in Figure 6b, fall outside the applicability of standard QSAR modeling. Nonetheless, VCs from SARMs representing discontinuous SAR regions are also attractive candidates for compound design. This especially applies to VCs falling into the vicinity of activity cliffs ^10^ that are formed by pairs of structural analogs with large potency differences, as illustrated in Figure 6b. Activity cliffs represent the pinnacle of SAR discontinuity. VCs in the vicinity of activity cliff can often be expected to display large (positive or negative) potency fluctuations and are hence attractive candidates in the search for potent hits. Although a QSAR formalism cannot be applied to predict the potency of such compounds, they can be easily selected from SARMs containing activity cliffs on the basis of a “guilt-by-association” principle, i.e., VCs are preferentially selected that are neighbors of potent activity cliff partners. For this purpose, SARMs capturing high degrees of local SAR discontinuity are selected on the basis of discontinuity ranking ( *vide supra*).

### Multi-target activity spaces

**Figure 7.**
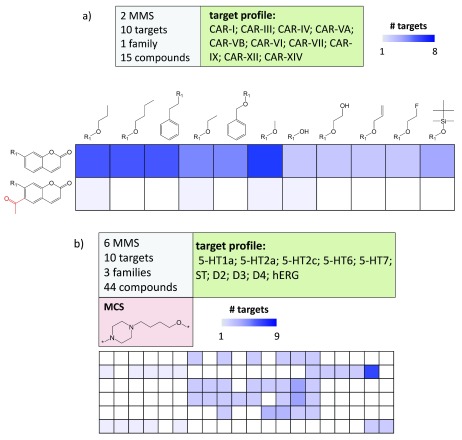
Multi-target compound series matrices. ( **a**) shows a CSM containing 15 inhibitors of 10 carbonic anhydrase (CAR) isoforms. Target coverage of analogs is reflected by increasingly dark blue shading of cells. Substructures distinguishing the core fragments are highlighted in red. The matrix composition is summarized (top left) and the target profile reported (top right). ( **b**) shows a CSM with 44 analogs active against 10 targets (including the hERG anti-target) belonging to three different families. The maximum common core structure (MCS) of the analog series is displayed. For clarity, compound structures are omitted. Target abbreviations: 5-HT; serotonin receptor, ST; serotonin transporter, D; dopamine receptor, hERG; hERG ion channel.

**Figure 8.**
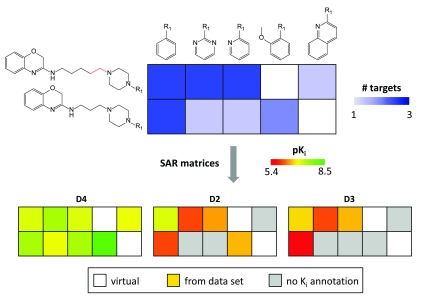
Matrix conversion. The deconvolution of a CSM with eight analogs active against the dopamine D2, D3, and D4 receptor isoforms into three single-target SARMs is illustrated. In all matrices, cells corresponding to VCs are not color-coded. In SARMs, cells of compounds with no available activity annotation for a given target are colored gray.

SARMs have also been adapted for the navigation of multi-target activity spaces, which are populated by promiscuous compounds. In this context, promiscuity is defined as the ability of a compound to specifically interact with multiple targets (as opposed to non-specific binding effects) ^11^. Here, the primary purpose of the matrix approach is not SAR analysis, but the systematic exploration of compound promiscuity patterns. Therefore, matrices capturing multi-target activities are generated. Such matrices have been designated as Compound Series Matrices (CSMs) ^6^. CSMs are of interest for chemogenomics applications in which compound-target interactions are systematically explored ^12^. In Figure 7, two exemplary CSMs of different composition and target coverage are shown that reveal different compound promiscuity patterns. In CSMs, data set compounds are color-coded according to the number of targets they are active against (instead of potency-based coloring). In Figure 7a, two structural analogs display very different degrees of promiscuity and in Figure 7b, a center of promiscuity is identified in a sparsely populated matrix. CSMs are designed to mine chemogenomics data sets and also offer immediate suggestions for the design of compounds with different multi-target activities. In addition, it is also readily possible to deconvolute CSMs into individual single-target SARMs, as illustrated in Figure 8. This makes it possible to compare SARMs across different targets and identify compounds that are attractive candidates for testing against additional targets.

### Programs and compounds

Java programs were written, in part with the aid of the OpenEye chemistry tool kit ^13^, to identify A_MMS and generate, rank, and display SARMs. Routines for potency predictions were also implemented in Java. Statistical analyses were carried out using R ^14^. All compounds shown herein were obtained from ChEMBL ^15^.

## Concluding remarks

Herein, we have reviewed the design of the SARM methodology and discussed recent extensions and selected applications. In-house implementations of the SARM approach have been continuously developed and further refined to increase the utility of the methodology for medicinal chemistry. Primary reasons for discussing the different aspects and applications of SARMs in context have been to expose this approach to a wider drug development audience and provide an example for the data- and application-driven evolution of a computational medicinal chemistry method. SARMs can essentially be rationalized as local activity landscapes of data sets that are based upon a unique and comprehensive structural organization. SARMs primarily focus on activity information associated with series of closely related compounds but can also be applied to systematically study compound promiscuity patterns. In addition, they can also be easily adapted to explore other structure-property relationships relevant to drug discovery. A special feature of SARMs that sets them apart from many other activity landscape representations is that they closely link descriptive compound data analysis (a primary task of activity landscape modeling) and prospective compound design. Because SARMs are reminiscent of conventional R-group tables, they are readily intuitive to medicinal chemists, thus circumventing the communication barrier that often hinders the effective application of computational approaches in the practice of medicinal chemistry. Future research activities will focus on the design of multi-property SARMs to aid in advanced compound optimization efforts.

## Data availability

The compound data sets used to generate the SARMs and the CSMs are available via ZENODO ^16^.
