# Supplementary material for: The ‘SAR Matrix’ method and its extensions for applications in medicinal chemistry and chemogenomics
Source: F1000Res. 2014 Jun 23;3:113. Originally published 2014 May 16. [Version 2] doi: 10.12688/f1000research.4185.2 (PMC4215758; doi:10.12688/f1000research.4185.2)
Supplement: Peer review history

# Peer review: Referee response for version 2

**DOI:** 10.5256/f1000research.4876.r5334

**Published:** 2014-07-02

**Authors:** Georgia B. McGaughey

**Affiliations:** 1 Vertex Pharmaceuticals Inc., Cambridge, MA, USA

Thank you for your responses. Revised manuscript looks good and I look forward to reading the paper in AAPS on off target effects.

I have read this submission. I believe that I have an appropriate level of expertise to confirm that it is of an acceptable scientific standard.

# Peer review: Referee response for version 1

**DOI:** 10.5256/f1000research.4481.r4991

**Published:** 2014-06-11

**Authors:** Georgia B. McGaughey, Jonathan Weiss

**Affiliations:** 1 Vertex Pharmaceuticals Inc., Cambridge, MA, USA

This is a well written article with a sound description of the Structure-Activity Relationship (SAR) Matrix (SARM) methodology. Only a few questions / suggestions are recommended.

1. In the Methods section, you describe the definition of a MMP. Specifically, you rely on the algorithm by Hussain and Rea. Have you considered MMPs where the only change is in a ring (i.e., an aromatic versus partially saturated ring)?
2. Continuing onwards in the Methods section, you extend the MMP concept to include Matched Molecular Series (MMS). While I believe you were the first to coin this description, there are now others who are also using this formalism (e.g. NextMove (Roger Sayle)) and some reference to these additional methods is advised, particularly to avoid confusion since the names are similar. If there are differences, perhaps you could expand on them.
3. In the Matrix distribution and ranking section, could you expand on the matrix overlap, row overlap and matrix coverage with at least one specific example (ie show the math in the supplementary material)? Additionally, I recommend using the same variables (i.e. are " *n*" and " *#real compounds*" the same)? If so, then they should be consolidated to one variable.  Additionally, it's not obvious how the numbers to the right (5%) and left (30%) of the two matrices are derived.
4. There appears to be some mis-counting of the number of targets in both Figure 7a & b. For Figure 7a, are there 10? And for Figure 7b, we count 10 targets and 4 families. Make sure to check the Figure 7 caption. Consider referring to DOP-D2 as merely D2; likewise DOP-D3 should be merely D3. Check the commas and semicolons as there are inconsistencies.
5. Can these multi-target compound series matrices be tied to Adverse Drug Reactions (ADR)?  That seems as a possible extension of this current work. In Figure 7b you consider hERG and hence, it seems as an opportunity to extend beyond merely the primary activity.
6. Consider changing the word " *accessible*" in the concluding remarks to "intuitive" or "interpretable".

We have read this submission. We believe that we have an appropriate level of expertise to confirm that it is of an acceptable scientific standard.

# Peer review: Referee response for version 1

**DOI:** 10.5256/f1000research.4481.r4803

**Published:** 2014-06-10

**Authors:** Herman van Vlijmen

**Affiliations:** 1 Janssen Infectious Diseases-Diagnostics BVBA, Beerse, Belgium

The manuscript on the SAR matrix method offers a useful approach to extract the relevant information from large datasets with compound activity data, and to present this in an intuitive way to chemists and computational chemists. It is therefore an attractive tool to use in analysis of HTS screens and to find structure activity trends and discontinuities in large groups of structurally related molecules.

The title and abstract cover the content well. The chemogenomics application of the method is related to the use of compound promiscuity instead of the more usual compound activity on a single target. The methods are clearly described and can most likely be reproduced. The datasets used (even the dataset from the public source CHEMBL) are not provided, so the results will be difficult to reproduce. There is no mention in the manuscript on the availability of the tools that were developed. Methods like these could get widespread usage if they would be available to a wider audience. It would also be good if at least the public dataset would be made available so the results can be compared to other approaches.

One significant benefit of this approach is that a large dataset can automatically be processed by the method to create multiple (often very many) SAR matrices. The authors point out that the idea is not that all these (often hundreds or thousands) SAR matrices are inspected visually, but that interesting elements in the matrices can be identified automatically, for instance "virtual compounds" (core-substituent combinations not yet made) that are predicted to have interesting activity. The same virtual compound can appear in different SAR matrices, and therefore multiple predictions can be made for the same compound and the level of consistency could be a good indicator for deciding to make he actual compound. The method also automatically identifies virtual compounds that are close to activity cliffs and are therefore interesting to make and test.

I have read this submission. I believe that I have an appropriate level of expertise to confirm that it is of an acceptable scientific standard.
